# Predictors of fear of childbirth and normal vaginal birth among Iranian postpartum women: a cross-sectional study

**DOI:** 10.1186/s12884-021-03790-w

**Published:** 2021-04-21

**Authors:** Forough Mortazavi, Maryam Mehrabadi

**Affiliations:** 1 Non-Communicable Diseases Research Center, Sabzevar University of Medical Sciences, Pardis Building, Towhidshahr Blvd, Sabzevar, Iran; 2 Health Chancellery, Sabzevar University of Medical Sciences, Sabzevar, Iran

**Keywords:** Fear of childbirth, Vaginal birth, Postpartum, Doula, Cesarean, Tokophobia

## Abstract

**Background:**

Fear of childbirth (FOC) may contribute to postpartum depression, impaired maternal-infant relation, and preference for cesarean in future pregnancies. We aimed to investigate predictors of FOC and normal vaginal birth among postpartum women who had planned for a normal vaginal birth.

**Methods:**

This cross-sectional study was conducted in 2019 with postpartum women during the first 24 h after the birth. A sample of 662 women, selected using a convenient sampling method, filled out the questionnaire composed of socio-demographic and obstetric questions and the Wijma Delivery-Expectancy Questionnaire (W-DEQ). We used multiple logistic regression analyses to determine predictors of FOC and normal vaginal birth.

**Results:**

The percentage of women with mild (score ≤ 37), moderate (38–65), high (66–84), severe (85–99), and intense FOC (score ≥ 100) were 7.9, 19.5, 40.9, 21.1, and 10.6% respectively. Predictors of intense FOC were age < 30, primiparity, low maternal satisfaction with pregnancy, and a low level of perceived marital satisfaction. Overall, 21.8% of women gave birth by cesarean. Predictors of normal vaginal birth were birth weight < 4 kg, spontaneous onset of labor pain, mother’s age < 30, term pregnancy, having a doula, multiparity, satisfaction with husband’s support, and overall satisfaction with pregnancy. A high level of perceived marital/sexual satisfaction was a risk factor for cesarean. Mode of birth was not a predictor of postpartum FOC.

**Conclusions:**

The rate of severe and intense FOC among this group of postpartum women is high. Our findings highlight modifiable factors for reducing FOC and increasing normal vaginal birth. In designing programs to increase the rate of normal vaginal birth, the following factors should be considered: limiting induced labor, encouraging women to recruit a doula to help them at labor, facilitate husband’s attendance throughout antenatal/intrapartum, and postnatal care to support his wife, and pay attention to women’s common misunderstandings about the effect of vaginal birth on marital/sexual relationship. Our findings indicate that seeking novel ways to promote marital/sexual satisfaction and helping women to have a smooth, hassle-free pregnancy may contribute to a reduction in the rate of the FOC.

**Supplementary Information:**

The online version contains supplementary material available at 10.1186/s12884-021-03790-w.

## Background

Most pregnant women regard childbirth as an important and challenging event which may be accompanied by fears and worries. Fear of childbirth (FOC) is common among pregnant women in Western countries with a prevalence rate of eight to 27% [1]. The prevalence of severe FOC in Iran was reported at 19.6% [2]. Several adverse consequences were reported in women with a high level of FOC including postpartum depression, impaired maternal-infant relation [3], preference for cesarean birth [4, 5], dystocia, and emergency caesarean birth [6].

Several studies have found that a number of factors might increase FOC including advanced maternal age, high socio-economic status [7], insufficient antenatal education [8], obstetric complications, increased analgesic use in labor [9], postdate pregnancy [10], low self-esteem [11], and low level of acceptance of pregnancy [5]. Earlier studies have shown that nulliparous women experience higher levels of fear than multiparous women before birth. However, recent studies indicate that there is no difference in levels of postpartum fear between these two groups [2, 6]. Lack of social support is also associated with FOC [12]. Pregnant women may receive support from family, spouse, and health care providers. Having a doula during pregnancy, birth, and postpartum may have a positive impact on maternal emotional well-being. It has been observed to reduce anxiety and stress and increase self-esteem and self-efficacy [13] and also reduce the cesarean rate [14].

Iran is among the countries which have a high rate of cesarean birth. Between 1976 and 2010, the overall incidence of caesarean increased from 19.5 to 48% in Iran [15] which led to the adoption of policies incorporated in the Health Transformation Plan (HTP) in 2014 to decrease the rate of caesarean birth [15]. These new policies involved a number of financial, infrastructural, and educational interventions including providing free hospital birth services and financial incentives for normal birth providers, improving the infrastructures of maternity facilities, and free childbirth preparatory classes. Pregnant women were also allowed to have their own midwife or a lay companion as a doula to accompany them during labor and birth [15, 16]. This was well received by pregnant women who planned to have a normal vaginal birth [17]. Studies have shown that since the implementation of the HTP, the rate of cesarean decreased significantly in public hospitals. But in the case of private hospitals, the policy was not successful and in fact their cesarean rates increased [18]. So, despite overall decline in cesarean rate in Iran after the implementation of the HTP, the intended goal of a 10% yearly reduction has not yet been achieved [19].

These findings from earlier studies suggest that it would be informative to investigate FOC among women who had planned for normal vaginal birth and who delivered via cesarean or normal vaginal birth after going into labor. Several questions are worth considering with regard to these women such as 1. Does severity of FOC differ between primiparous and multiparas? 2. Do primiparous and multiparas differ with respect to different dimensions of FOC like fear of loss of baby or loneliness? 3. Does severity of FOC differ between women who had a doula at birth and those who had not? 4. Is there a relationship between FOC and mode of birth? 5. What are predictors of FOC and normal vaginal birth in women who planned for a normal vaginal birth?

In designing policies to reduce FOC and cesarean rate, it is important to identify predictors of FOC and normal vaginal birth among postpartum women. Most of the previous studies about FOC have focused on evaluating antenatal FOC by investigate women’s expectations towards childbirth. In contrast, we aimed to investigate FOC in early postpartum to explore women’s actual experience of childbirth among those who had planned for normal vaginal birth.

## Methods

This study was conducted using data previously collected for a descriptive cross-sectional study on the validity of the Persian Birth Satisfaction Scale-Revised (the Persian BSS-R) [20]. The Ethics Committee of Sabzevar University of Medical Sciences has approved this study (Number: IR.MEDSAB.REC.1399.119). All methods were performed in accordance with guidelines of the Sabzevar University which is in accordance with the Declaration of Helsinki**.** The study population was postpartum women. Mothers hospitalized in the postpartum wards of Mobini Hospital, affiliated with Sabzevar University of Medical Sciences, Sabzevar, Iran were recruited during the first 24 h after birth using a convenience sampling method. Recruitment for this study began in July and ended in September 2019. Before the COVID-19 pandemic, there were on average 6000 births in this hospital annually.

The inclusion criteria for the validity study of the questionnaire were having a pregnancy with a healthy single baby, ability to read and write, being physically able to fill out the questionnaires and giving consent to participate in the study. Exclusion criteria included mental illness requiring medication and having postpartum complications such as fever, severe hemorrhage, high blood pressure, or any other complications that may have compromised the accuracy of responses to the items of Wijma scale. For the present analysis, we excluded women who planned elective cesarean as well as those with vaginal birth after cesarean (VBAC) and instrumental birth.

We instructed two graduate midwives on data collection. After obtaining verbal consent from participants in the study, the midwives extracted obstetrical information from the patients’ files, distributed the written consent forms and the questionnaires among all postpartum women, and instructed them on how to fill out the anonymous questionnaires.

### Instruments

#### Interview form

The women were interviewed at the postpartum and an interview form was completed. It consisted of three sections containing questions on socio-demographic characteristics (such as age, level of education, employment status, monthly family income), obstetrical information (such as parity, mode of birth, having a spontaneous onset of labor pain, pain relief method during labor, having a doula at birth, infant birth weight), and psychosocial factors (i.e., the level of satisfaction with pregnancy, husband’s support, and marital/sexual relationship). We instructed the women to rate their level of satisfaction with pregnancy, husband’s support, and marital/sexual relationship on a five-point Likert scale ranging from one (not satisfied) to five (very satisfied). With regard to this issues, we asked the following questions: 1. To what extend are you satisfied with your pregnancy, given the health problems you encountered during your pregnancy?, 2. To what extent are you satisfied with your husband’s emotional/financial support during pregnancy?, and 3. To what extent are you satisfied with your marital/sexual relationship during the pregnancy? We asked women to rate their satisfaction with monthly household income on a three-point Likert scale ranging from 1 indicating a low level of satisfaction, 2 indicating satisfaction, and 3 indicating a high level of satisfaction (the supplementary file).

#### Wijma delivery expectancy/experience questionnaire (W-DEQ) version B

The Wijma Delivery Expectancy/Experience Questionnaires (W-DEQ) was developed to investigate postpartum FOC [21]. The W-DEQ is unidimensional and contains 33 items that are rated on a six-point Likert scale ranging from zero (strongly disagree) to five (strongly agree). The minimum and maximum total scores of the scale are 0 and 165, respectively, with higher scores indicating higher fear. In addition, Wijma et al. proposed two cut-off points of 85 and 100 for screening women with clinical, severe childbirth fear. In the next study, Toohill, et al. proposed scores ≤37 as mild fear, 38–65 as moderate fear, 66–84 as high fear, and ≥ 85 as severe fear [1]. The reliability of the scale was excellent (Cronbach’s alpha = 0.93). The validity of the W-DEQ was confirmed by the moderate correlations between the scale and several psychological scales [21]. The W-DEQ was translated into Persian. The Persian W-DEQ which consists of six factors, showed moderate correlation with the Childbirth Attitude Questionnaire and the State-Trait Anxiety Inventory (STAI). The Cronbach’s alpha coefficients of the scale and its factors were in the acceptable range (between 0.633 and 0.919) [22]. In this study, we examined both cut-off points for Wijma scores (85 and 100) for screening women with FOC.

### Data analysis

SPSS version 18 was used to analyze the data. Descriptive statistics were used to define the sample characteristics. Normal distribution of the Wijma scores was confirmed using skewness or kurtosis. The women were classified into two groups according to parity and the mean scores of W-DEQ for the two groups were compared using t-test. We investigated the relationship between having a doula and FOC in cesarean and vaginal birth using two t-test, one in the normal vaginal birth group and the other in the cesarean group. Multiple logistic regression analyses by backward-LR method was used to determine independent variables predicting the mode of birth and FOC. All variables with a *p*-value < 0.25 in simple logistic regression analysis were entered into the multiple logistic regression analysis.

## Results

Of the 784 participants who were recruited for the validity study, we removed cases who had chosen elective cesarean (*n* = 101), VBAC, and instrumental vaginal birth (*n* = 21); so, the sample size for the present study is 662. The mode of birth for 21.8% of women was emergency cesarean; the corresponding percentages among primiparous and multiparous were 24 and 20.1%, respectively. The percentage of primiparous and multiparous who had a doula at birth were 31.1 and 25.6%, respectively. Participants’ demographic and obstetric characteristics is presented in Table 1.

**Table 1:** Participants’ demographic and obstetric characteristics (*N* = 662)

Demographic/obstetric Variables	Mean ± SD	N (%)
Age (years)	28.1 ± 6.2	
Educational level (years)	11.0 ± 3.6	
Gestational age at birth (week)	39.32 ± 1.2	
Birth weight (gr) Mean ± SD	3163.7 ± 487.4	
Job		
Housewife		603 (91.1)
Employed		59 (8.9)
Satisfaction with household income		
Low satisfied		239 (36.1)
Moderately satisfied/satisfied		423 (63.9)
Parity		
Primipara		283 (42.7)
Multipara		379 (57.3)
Mode of birth		
Emergency cesarean		144 (21.8)
Vaginal birth		518 (78.2)

The means of the W-DEQ total scores and the mean scores of all its six factors for primiparous and multiparous women are presented in Table 2. The means of the loneliness, fear, and loss of control factors and the mean of the W-DEQ total score are higher in primiparous than multiparas (*p* <  0.05). There was a significant relationship between parity and levels of FOC (*p* <  0.008).

**Table 2:** Fear of childbirth according to parity (*N* = 662)

	All	Primiparous (*N* = 283)	Multiparas (*N* = 379)	t	*P*
Domains of fear of childbirth	Mean ± SD	Mean ± SD	Mean ± SD		
Lack of self-efficacy	23.7 ± 11.7	24.2 ± 12.6	23.3 ± 10.9	0.908	0.374
Lack of positive anticipation	5.4 ± 4.1	5.7 ± 4.1	5.2 ± 4.0	1.432	0.153
Concerns for fetus health	3.1 ± 3.5	3.2 ± 3.4	3.1 ± 3.5	0.356	0.722
Loss of control	5.3 ± 3.2	5.6 ± 3.2	5.1 ± 3.1	2.129	0.034*
Loneliness	18.9 ± 9.4	19.9 ± 9.0	18.2 ± 9.6	2.336	0.020*
Fear	15.4 ± 5.2	16.1 ± 5.1	14.9 ± 5.2	2.973	0.003**
Total score	74.7 ± 23.1	77.5 ± 24.3	72.5 ± 21.9	2.766	0.006**
	N (%)	N (%)	N (%)		
Fear of childbirth				13.72	0.008**
Mild (scores ≤37)	52 (7.9)	23 (8.1)	29 (7.7)		
Moderate (38 ≤ scores < 66)	129 (19.5)	45 (15.9)	84 (22.2)		
High (66 ≤ scores < 85)	271 (40.9)	116 (41.0)	155 (40.9)		
Severe (85 ≤ scores < 100)	140 (21.1)	56 (19.8)	84 (22.2)		
Intense (scores ≥100)	70 (10.6)	43 (15.2)	27 (7.1)		

*p < 0.05, **p < 0.01

There was a significant relationship between severe FOC (W-DEQ ≥ 85) and having a doula at childbirth (*p* = 0.047). We found no significant relationship between severe FOC (W-DEQ > 85) and mode of birth (*p* = 0.092) (Table 3).

**Table 3:** Predictors of severe fear of childbirth (Wijma score ≥ 85)

Variables	Simple logistic regression			Multiple logistic regression			
	score < 85	score ≥ 85	OR	*p*	AOR	95% C.I for OR	
						Lower	Upper
Age (years)							
< 30	253 (56.0)	132 (62.9)	1.33^†^	.048	1.428	1.004	2.033
> 30	199 (44.0)	78 (37.1)	1	–	1	–	–
Educational level (years)							
< 12	341 (75.4)	171 (81.4)	1.43^†^	–	–	–	–
> 12	111 (24.6)	39 (18.6)	1	–	–	–	–
Satisfaction with household income							
Low satisfied	147 (32.5)	92 (43.8)	1.62**	–	–	–	–
Moderately satisfied/satisfied	305 (67.5)	118 (56.2)	1	–	–	–	–
Gestational age (week)							
< 38	64 (14.2)	26 (12.4)	1.07	–	–	–	–
38–40	322 (71.2)	159 (75.7)	1.30	–	–	–	–
> 40	66 (14.6)	25 (11.9)	1	–	–	–	–
Parity							
Primipara	184 (49.7)	99 (47.1)	1.30^†^	–	–	–	–
Multipara	268 (59.3)	111 (52.9)	1	–	–	–	–
Birth mode							
Cesarean	90 (19.9)	54 (25.7)	1.39^†^	–	–	–	–
Normal vaginal birth	362 (80.1)	156 (74.3)	1	–	–	–	–
Having a doula							
No	315 (69.7)	162 (77.1)	1.47*	–	–	–	–
Yes	139 (30.3)	65 (22.9)	1	–	–	–	–
Onset of labor pain							
Spontaneous	313 (69.2)	145 (69.0)	1	–	–	–	–
Induced	139 (30.8)	65 (31.0)	0.98	–	–	–	–
Pain relief method							
Entonox	226 (50)	93 (44.3)	.86	–	–	–	–
Spinal anesthesia	85 (19.7)	50 (25.7)	1.27	–	–	–	–
Hot water showers or massage	18 (4.0)	6 (2.9)	.70	–	–	–	–
Nothing	119 (26.3)	57 (27.1)	1	–	–	–	–
Satisfaction with pregnancy							
Not satisfied	13 (2.9)	24 (11.4)	16.62***	<.001	13.868	4.631	41.528
Low satisfied	33 (7.3)	33 (15.7)	9.0***	<.001	7.072	2.628	19.030
Moderately satisfied	166 (36.7)	76 (36.2)	4.1**	.016	3.047	1.230	7.549
Satisfied	186 (41.2)	71 (33.8)	3.4**	.029	2.732	1.110	6.723
Very satisfied	54 (11.9)	6 (2.9)	1	–	1	–	–
Perceived marital/sexual satisfaction							
Not satisfied to moderately satisfied	39 (8.6)	29 (13.8)	2.59**	.018	2.066	1.135	3.762
Satisfied	218 (48.2)	125 (59.5)	2.00***	.001	1.890	1.280	2.792
Very satisfied	195 (43.1)	56 (26.7)	1	–	1	–	–
Satisfaction with husband’s support							
Not satisfied to moderately satisfied	81 (17.7)	62 (29.5)	2.06**	–	–	–	–
Satisfied	212 (46.9)	89 (42.4)	1.13	–	–	–	–
Very satisfied	159 (35.2)	59 (28.1)	1	–	–	–	–

^†^simple logistic regression: *p* < 0.25, *p < 0.05, **p < 0.01, ***p < 0.001, Variables entered on step 1: education, satisfaction with income, parity, mode of birth, having a doula, maternal satisfaction with pregnancy, perceived marital/sexual satisfaction, and satisfaction with husband support. method: forward LR, Cox & Snell R Square = 8.7%, Nagelkerke R Square = 12.1%

Correlates of severe FOC (Wijma score ≥ 85) include low satisfaction with household income, not having a doula at birth, low level of satisfaction with pregnancy, low level of marital/sexual satisfaction, and low level of satisfaction with husband’s support. We conducted multiple logistic regression analysis on Wijma scores to determine socio-demographic/obstetric and psychological predictors of severe FOC (Wijma score ≥ 85). Age < 30, low level of satisfaction with marital/sexual relationship and low level of satisfaction with pregnancy predicted severe FOC (Table 3).

Correlates of intense FOC (Wijma score ≥ 100) include age < 30, primiparity, low level of satisfaction with pregnancy, and low perceived quality of marital/sexual relationship. We conducted multiple logistic regression analysis on Wijma scores to determine predictors of intense FOC (score ≥ 100). Age < 30, primiparity, low level of satisfaction with pregnancy, and low level of perceived marital/sexual satisfaction predicted intense FOC (Table 4).

**Table 4:** Predictors of intense fear of childbirth (Wijma score ≥ 100)

Variables	Simple logistic regression			Multiple logistic regression			
	score < 100	score ≥ 100	OR	*p*	AOR	95% C.I for OR	
Age (years)						Lower	Upper
< 20	40 (6.8)	8 (11.4)	2.88**	.079	2.58	.89	7.42
20–30	293 (49.5)	44 (62.9)	2.16*	.040	1.99	1.03	3.85
> 30	259 (43.8)	18 (25.7)	1		1		
Educational level (years)							
< 12	457 (77.2)	55 (78.6)	1.08	–	–	–	–
> 12	135 (22.8)	15 (21.4)	1	–	–	–	–
Satisfaction with household income							
Low satisfied	208 (35.1)	31 (44.3)	1.47^†^	–	–	–	–
Moderately satisfied/satisfied	384 (64.9)	39 (55.7)	1	–	–	–	–
Gestational age (week)							
< 38	83 (14.0)	7 (10.0)	.77	–	–	–	–
38–40	427 (72.1)	54 (77.1)	1.15	–	–	–	–
> 40	82 (13.9)	9 (12.9)	1	–	–	–	–
Parity							
Primipara	240 (40.5)	43 (61.4)	2.34**	.035	1.94	1.05	3.60
Multipara	352 (59.5)	27 (38.6)	1		1		
Birth mode							
Cesarean	127 (21.5)	17 (24.3)	1.17	–	–	–	–
Normal vaginal birth	465 (78.5)	53 (75.5)	1	–	–	–	–
Having a doula							
No	423 (71.5)	54 (77.1)	1.35	–	–	–	–
Yes	169 (28.5)	16 (22.9)	1	–	–	–	–
Onset of labor pain							
Spontaneous	410 (69.3)	48 (68.6)	1	–	–	–	–
Induced	182 (30.7)	22 (31.4)	1.07	–	–	–	–
Pain relief method							
Entonox	281 (48.1)	38 (54.3)	1.56	–	–	–	–
Spinal anesthesia	120 (20.5)	15 (21.4)	1.36	–	–	–	–
Hot water showers or massage	21 (3.6)	3 (4.3)	1.65	–	–	–	–
Nothing	162 (27.7)	14 (20.0)	1	–	–	–	–
Satisfaction with pregnancy							
Not satisfied	21 (3.5)	16 (22.9)	44.95***	<.001	12.14	5.17	28.50
Low satisfied	53 (9.0)	13 (18.6)	14.47*	<.001	4.94	2.19	11.10
Moderately satisfied	218 (36.8)	24 (34.3)	6.50^†^	.078	1.83	.935	3.57
Satisfied/Very satisfied	300 (50.7)	17 (24.3)	3.92^†^		1		
Perceived marital/sexual satisfaction							
Not satisfied to moderately satisfied	54 (9.1)	14 (20.0)	2.70**	.018	2.68	1.18	6.09
Satisfied	309 (52.2)	34 (48.6)	1.15	.612	1.17	.639	2.14
Very satisfied	229 (38.7)	22 (31.4)	1		1		
Satisfaction with husband support							
Not satisfied to low satisfied	17 (2.9)	4 (5.7)	2.10^†^	–	–	–	–
Moderately satisfied	104 (17.6)	18 (25.7)	1.54^†^	–	–	–	–
Satisfied	275 (46.5)	26 (37.1)	.84	–	–	–	–
Very satisfied	196 (33.1)	22 (31.4)	1	–	–	–	–

^†^simple logistic regression: *p* < 0.25, *p < 0.05, **p < 0.01, ***p < 0.001, Variables entered on step 1: age, satisfaction with income, parity, maternal satisfaction with pregnancy, perceived marital/sexual satisfaction, and satisfaction with husband support. method: backward LR, Cox & Snell R Square = 8.9%, Nagelkerke R Square = 18.2%

To investigate whether the relationship between having a doula and FOC is influenced by mode of birth, we conducted two t-test, one in the normal vaginal birth group and the other in the cesarean group. In the normal vaginal birth group, women who had a doula for childbirth experienced a lower level of fear than those who did not have a doula (*p* < 0.001). In the cesarean group, we found no significant difference between the mean scores of W-DEQ in women who had a doula at birth and those who had not (*p* = 0.117) (Table 5).

**Table 5:** Distribution of Wijma scores according to the mode of birth and having a Doula at birth

Having a doula at birth	Emergency cesarean		Vaginal birth	
	N	Mean ± SD	N	Mean ± SD
No	129	80.2 ± 18.1	348	75.6 ± 23.2
Yes	15	86.9 ± 16.4	170	67.4 ± 25.0
t	1.36		3.69	
*P*	0.117		< 0.001	

Our results indicate that in the case of women who planned for normal vaginal birth, seven factors influenced the final mode of birth. Cesarean was more prevalent among women with the following characteristics: age > 30, having induced labor, not having a doula at birth, gestational age < 38 week, and birth weight > 4 kg or < 2.5 kg. Women who were satisfied with their husband’s support and those who were satisfied with their pregnancy were more likely to give birth by normal vaginal birth. Satisfaction with marital/sexual relationship was a protective factor for vaginal birth [*p* = 0.015, OR = .374, CI (.169, .827)]. We entered 10 variables with *p*-value < 0.25 into a multiple logistic regression analysis. Nine variables remained in the model (Table 6).

**Table 6:** Predicting factors of normal vaginal birth in women who planned for normal vaginal birth (*N* = 662)

Variables	Simple logistic regression			Multiple logistic regression			
	Normal vaginal birth (*N* = 478)	Emergency Cesarean (*N* = 144)	OR	*P*	AOR	95% C.I for OR	
						Lower	Upper
Age (years)							
< 20	37 (7.1)	11 (7.6)	1.27	.082	2.157	.906	5.137
20–30	280 (54.1)	57 (39.6)	1.86**	<.001	2.396	1.469	3.909
> 30	201 (38.8)	76 (52.8)	1		1		
Educational level (years)							
< 12	109 (75.7)	403 (77.8)	1.125	–	–	–	–
> 12	35 (24.3)	115 (22.2)	1	–	–	–	–
Job							
Housewife	472 (91.1)	131 (91.0)	1.02	–	–	–	–
Employed	46 (8.9)	13 (9.0)	1	–	–	–	–
Satisfaction with household income							
Low satisfied	180 (34.7)	59 (41.0)	1	–	–	–	–
Moderately satisfied/satisfied	338 (65.3)	85 (59.3)	1.30^†^	–	–	–	–
Gestational age (week)							
< 38	51 (9.8)	39 (27.1)	1		1		
38–40	391 (75.5)	90 (62,5)	3.22***	<.001	3.244	1.818	5.788
> 40	76 (14.7)	15 (10.4)	3.87***	.001	3.801	1.685	8.577
Birth weight (gr)							
< 2500 g	42 (8.1)	18 (12.5)	1.50	.007	5.008	1.556	16.123
2500–3999	462 (89.2)	117 (81.3)	2.54*	.007	3.657	1.416	9.446
> 4000	14 (2.7)	9 (6.3)	1		1		
Having a doula							
No	348 (67.2)	129 (89.6)	1		1		
Yes	170 (32.8)	15 (10.4)	4.20***	<.001	4.419	2.420	8.068
Parity							
Primipara	215 (41.5)	68 (47.2)	1		1		
Multipara	303 (58.5)	76 (52.8)	1.26†	.010	1.937	1.175	3.192
Infant gender							
Female	268 (51.7)	80 (55.6)	1	–	–	–	–
Male	250 (48.3)	64 (44.4)	1.17	–	–	–	–
Onset of labor pain							
Spontaneous	375 (72.4)	83 (57.6)	1.72**	.030	1.624	1.049	2.515
Induced	143 (27.6)	61 (43.4)	1		1		
Satisfaction with husband support							
Not satisfied to low satisfied	99 (19.1)	44 (30.6)	1		1		
Moderately satisfied	243 (46.9)	58 (40.3)	1.86**	.001	2.543	1.475	4.387
Satisfied/Very satisfied	176 (34.0)	42 (29.1)	1.86*	.063	2.024	.964	4.251
Perceived marital/sexual satisfaction							
Not satisfied to moderately satisfied	56 (10.8)	12 (8.3)	1	–	1	–	–
Satisfied	260 (50.2)	83 (57.6)	.67^†^	.015	.374	.169	.827
Very satisfied	202 (39.0)	49 (34.0)	.88	.076	.423	.164	1.096
Satisfaction with pregnancy							
Not satisfied/ Low satisfied	67 (12.9)	36 (25.0)	1		1		
Moderately satisfied	190 (36.7)	52 (36.1)	1.46	.345	1.547	.626	3.827
Satisfied	204 (39.4)	53 (36.8)	2.49*	.042	2.285	1.031	5.063
Very satisfied	57 (11)	3 (2.1)	3.18**	.012	2.741	1.248	6.020

*p < .05, ** *p* < .01, ***p < .001, ^†^simple logistic regression: *p* < 0.25

Variables entered on step 1: having a doula, income, parity, birth weight, gestational age, mother’s age, onset of labor pain, maternal satisfaction with pregnancy, satisfaction with husband support, satisfaction with marital/sexual relationship. Method: backward, LR Cox & Snell R Square = 14.5%, Nagelkerke R Square = 22.4%

## Discussion

We investigated the predictors of FOC and normal vaginal birth. Our results show that overall, primiparous women had a higher FOC scores than multiparas. Also, the percentages of women experiencing intense FOC were higher in primiparous than multiparas. In our previous study on pregnant women, levels of FOC were not different between nulliparous and multiparous women [2]. In the study of Toohill et al. in Australia, 31.5% of nulliparous and 18% of multiparous pregnant women reported high levels of fear [1]. Further investigation revealed that scores for the two factors of feeling lonely and being concerned about loss of control were higher among primiparous than multiparas but the two groups did not differ with regard to perceived lack of self-efficacy, lack of positive anticipation, and concerns for fetus health. The preceding points about the different domains of FOC should be taken into account in designing educational programs for reducing FOC in primiparous women.

The percentage of women with high (66–84), severe (85–99), and intense FOC (score ≥ 100) were 40.9 and 21.1%, and 10.6%, respectively. The above percentages are higher compared to those obtained in our previous study on pregnant women indicating that FOC is more prevalent in early postpartum. In that study, the prevalence of severe and intense antepartum FOC were 19.6 and 6.1%, respectively [2]. These results are not in agreement with the results of a study in Malawi which had found that the prevalence high FOC in pregnant women was twice the corresponding rate in postpartum women [23]. The prevalence of severe FOC in Indian women in the postpartum period was 13.1% [24].

The rates of prevalence of high and severe FOC in our study are generally higher than those reported in western countries [1]. In a study in Ireland, the prevalence of high and severe FOC were 36.7 and 5.3%, respectively [25]. In the study of Storksen et al. in Norway, 8 % of the women had severe FOC [26]. In the study of Toohill et al. in Australia, the prevalence of high FOC was 24% [1]. One factor which might explain the difference between our results and those of other studies is that we measured FOC during the 24 h after birth while other studies were conducted during pregnancy or with a period after giving birth. In addition, variation in instruments used to measure fear of birth should be considered. Furthermore, different birth conditions may have a role in explaining the difference between our results and those of other studies. We conducted our study in a maternity hospital where women undergoing labor share the same space with other parturient women while in western countries, maternity hospitals usually have birth suite where a parturient women stays is in a separate room with her family during her stay in the hospital. A recent qualitative study found that certain factors act as a barrier and reduce women’s demand for vaginal birth. These include perceived sub-optimal quality of care during labor and birth, limited physical space in maternity wards, and lack of privacy and dignity [27].

Predictors of intense FOC were mothers’ age < 30, primiparity, low level of satisfaction with pregnancy, and low level of perceived marital/sexual satisfaction. Primiparity has been found to be associated with antenatal FOC in several studies [1, 6, 28, 29]; however, such an association was not observed in a study conducted on postpartum women [6]. Although one study indicated that satisfaction with marital life was not associated with FOC [30], another study found that marital relationship could predict pregnancy anxiety [31] which is in turn strongly correlated with FOC according to several studies [26, 29, 30, 32]. In a study conducted in Istanbul, there were significant but weak correlations between FOC scores and the two factors of being pleased with pregnancy and accepting the motherhood role [5]. Further studies are needed to investigate if a low level of perceived marital/sexual satisfaction have a role in FOC.

Satisfaction with husband’s support was a correlate of FOC in our study. Some studies investigated the association of different sources of support and FOC. Women’s satisfaction with husband’s support [32], family support [11], intrapartum support [28], informational support [25], and couple adjustment [29] have been found to be predictors of FOC in previous studies.

Our results show that having a doula at birth could reduce FOC in women who gave birth by normal vaginal birth. This result is in agreement with a previous study which found that doula support reduced anxiety and tension and had a positive impact on maternal emotional wellbeing [13]. In contrast, women who had a doula at birth but finally gave birth by cesarean, experienced the same level of fear as those who did not have a doula and gave birth by cesarean. In recent years, childbirth preparatory classes have become increasingly popular among pregnant women. Participants in such classes may opt to have a doula during labor. It seems that women who choose to have a doula to give birth by normal vaginal birth but fail to deliver normally, experience frustration as a consequence. The results of a study revealed that a mismatch between a woman’s preferred mode of birth and actual mode increases the risk of developing post-traumatic stress symptoms [33]. Such cases should be taken into account in designing educational programs for reducing FOC.

Our results indicate that there is no association between mode of birth and FOC. This may be because our sample consisted of women who planned to have normal birth. Women with high or severe fear of childbirth might opt for an elective cesarean. Also, we excluded instrumental births which numbered only 21. This group’s experience of childbirth is painful and difficult and so it may provoke severe fear. In contrast to this, in Fenwick et al. study, cesarean increased levels of postpartum fear [6].

Overall, 78.2% of women in our study experienced a normal vaginal birth. According to our results, demographic and obstetric predictors of normal vaginal birth were birth weight < 4 kg, spontaneous onset of labor pain, mother’s age < 30, term pregnancy, having a doula, and multiparity. According to a study on 284 Nigerian nulliparous women in which 74.8% of the parturient gave birth by vaginal birth, normal infant birth weight was a factor associated with vaginal birth [34]. In the study of Prosser et al. in which 28.7% of women had a normal birth, predictors of normal vaginal birth were multiparity, younger age, spontaneous labor, lower gestational age, and knowing the midwives before labor and childbirth [35].

There were also two psychological predictors of normal vaginal birth, namely satisfaction with husband’s support and satisfaction with pregnancy. This implies that women with a hassle free pregnancy and those with a supportive husband are more likely to give birth normally. Satisfaction with marital/sexual relationship was a protective factor for vaginal birth. This means that women who were satisfied with their marital/sexual relationships were more likely to give birth by cesarean than those with a low level of marital/sexual satisfaction. It is a common belief that vaginal birth may affect sexual pleasure/function and consequently marital relationship. The results of a recent qualitative study in Iran indicates that fear of irreversible damage to urogenital organs and sexual function and husbands’ concerns about sexual function were among the reasons for requesting cesarean [36].

The high cesarean rates during recent decades in Iran are both a symptom and a cause of changes in community norms resulting in maternal requests for cesarean. Affluent women usually opt for elective cesarean in luxury hospitals. Some women want their baby to be born on a particular day and some are concerned about the function of their urogenital organs and sexual satisfaction after vaginal birth, and there are women who believe that elective cesarean is safer for the fetus [37]. Other reasons of requesting an elective cesarean are: fear of labor pain and vaginal birth, cultural attitudes associating cesarean with higher social status, considering husband’s payment of high costs of cesarean as a token of love and support, and influence of media, family, friends, doctors, and health professionals [36].

This study has a number of limitations. First, sampling was performed in a maternity hospital during the first 24 h after birth. In early postpartum, mothers may still feel uncomfortable and it can cause false negative/positive results. The second limitation was that we had to exclude women who had instrumental birth because we could not compare this small group with the normal vaginal birth group.

One of the strengths of this study is that we recruited a relatively large sample of women who planned to have normal birth. Also, we used the W-DEQ version B to measure the severity of postpartum FOC. The W-DEQ provides information on different domains of the fear. One of the disadvantages of this study was that we did not use validated scales to measure the levels of husband’s support, perceived marital/sexual satisfaction, and mothers’ satisfaction with pregnancy. Because all these factors have significant association with FOC and also with vaginal birth, we recommend that further research be conducted on postpartum FOC using valid scales to measure these variables. Also, further studies are needed to investigate women’s concerns about the negative effects of vaginal birth on marital/sexual relation and its association with FOC.

In this study, we tested both cut-off points for Wijma scores and obtained quite similar results with regard to predictors of FOC. The use of cut-off point ≥85 compared with cut-off point ≥100 yielded more variables that were correlated with severe FOC.

## Conclusions

Intense FOC is more prevalent among primiparous than multiparas. Inspection of factors likely to influence FOC showed that the mode of birth did not have a significant effect on FOC. In contrast, psychological variables such as mother’s satisfaction with pregnancy or her satisfaction with marital/sexual relationship could predict FOC. So, in designing programs for reducing FOC, researchers and policy makers in Iran should pay more attention to psychological factors.

The prevalence of high, severe, and intense FOC cases in our study are higher in comparison with those reported in western countries. We also found that postpartum FOC was not influenced by the mode of birth. Therefore, to reduce FOC health policymakers should learn from the experience of countries with low levels of FOC and adopt measures such as improving birth conditions, offering more choice to women with respect to labor and birth, avoiding unnecessary interventions during childbirth, promoting normal physiologic birth and respectful maternity care. Within the framework of the HTP, more emphasis should be placed on respectful maternity care and humanizing maternity services.

According to our results, two psychological variables could predict normal vaginal birth namely satisfaction with husband’s support and women’s satisfaction with pregnancy. This means that these variables could be manipulated to reduce cesarean. We also found that satisfaction with marital/sexual relationship was a protective factor for vaginal birth. Therefore, we recommend that interventional studies to reduce the rate of cesarean pay attention to women’s common misunderstandings about the effects of vaginal birth on sexual/marital relationship.

Our findings highlight factors which could be modified to increase normal birth. Limiting induced labor, encouraging women to have a doula to help them at labor, facilitating husbands’ attendance throughout antenatal/intrapartum and postnatal care to support their wives, consultation with couples to increase husband’s support, and attempts to make pregnancy safe and hassle-free should be considered in programs to increase the rate of normal vaginal birth.

## Supplementary Information

**Additional file 1.**

## Data Availability

The data that support the findings of this study are available from the corresponding author upon reasonable request.

## Abbreviations

FOC: Fear of childbirth
HTP: Health transformation plan
W-DEQ: The Wijma Delivery Expectancy/Experience Questionnaires
