# Supplementary material for: Predictors of fear of childbirth and normal vaginal birth among Iranian postpartum women: a cross-sectional study
Source: BMC Pregnancy Childbirth. 2021 Apr 21;21:316. doi: 10.1186/s12884-021-03790-w (PMC8058756; doi:10.1186/s12884-021-03790-w)
Supplement: Supplementary file 1 — Additional file 1. [file 12884_2021_3790_MOESM1_ESM.docx]

**Predictors of fear of childbirth and normal vaginal birth among Iranian postpartum women: a cross-sectional study**

Code:

Dear colleague. Please collect data from the participant’s file carefully.

1. Gestational age at the time of admission ………… week
2. Infant birth weight ………………………… gram
3. How many times has she given birth to a child? Parity = ……
4. How many times has she been pregnant? Gravidity = ……
5. What is the participant’s mode of delivery?
6. Elective cesarean b. emergency cesarean

c. normal vaginal birth d. VBAC e. vacuum delivery

1. Was the labor spontaneous or induced?
2. Spontaneous b. induced c. elective cesarean
3. How long did it take from admission to hospital to giving birth? ………… hours
4. Which analgesic method did she use in labor?
5. Entonox b. massage c. taking a shower

d. nothing e. epidoural/spinal

Dear mother. Please response to each item or question carefully.

1. How old are you? ………………. years
2. How many years have you studied? ……………….. years
3. To what extend are you satisfied with your household income?
4. low level of satisfaction b. satisfaction c. high level of satisfaction
5. What is your job?
6. Housewife b. employed
7. What is your infant gender?
8. Male b. Female
9. Did you have a doula at birth?
10. No b. yes
11. To what extend are you satisfied with your pregnancy, given the health problems you encountered during your pregnancy?
12. Not at all b. low satisfied c. moderately satisfied
13. Satisfied e. very satisfied
14. To what extent are you satisfied with your husband’s emotional/financial support?
15. Not at all b. low satisfied c. moderately satisfied
16. Satisfied e. very satisfied
17. To what extent are you satisfied with your marital/sexual relation?
18. Not at all b. low satisfied c. moderately satisfied
19. Satisfied e. very satisfied
